# *In vivo* Population Averaged Stereotaxic T2w MRI Brain Template for the Adult Yucatan Micropig

**DOI:** 10.3389/fnana.2020.599701

**Published:** 2020-11-13

**Authors:** Stephano J. Chang, Andrea J. Santamaria, Francisco J. Sanchez, Luz M. Villamil, Pedro Pinheiro Saraiva, Jose Rodriguez, Yohjans Nunez-Gomez, Ioan Opris, Juan P. Solano, James D. Guest, Brian R. Noga

**Affiliations:** ^1^Neuroscience Graduate Program, University of Miami Miller School of Medicine, Miami, FL, United States; ^2^The Miami Project to Cure Paralysis, University of Miami Miller School of Medicine, Miami, FL, United States; ^3^Division of Neurosurgery, Department of Surgery, University of British Columbia, Vancouver, BC, Canada; ^4^Interdisciplinary Stem Cell Institute, University of Miami Miller School of Medicine, Miami, FL, United States; ^5^Department of Pediatric Critical Care, University of Miami Miller School of Medicine, Miami, FL, United States; ^6^Department of Neurological Surgery, University of Miami Miller School of Medicine, Miami, FL, United States

**Keywords:** brain, template, MRI, stereotaxic, pig, segmentation, image analysis, population

## Abstract

Population averaged brain templates are an essential tool for imaging-based neuroscience research, providing investigators with information about the expected size and morphology of brain structures and the spatial relationships between them, within a demographic cross-section. This allows for a standardized comparison of neuroimaging data between subjects and provides neuroimaging software with a probabilistic framework upon which further processing and analysis can be based. Many different templates have been created to represent specific study populations and made publicly available for human and animal research. An increasingly studied animal model in the neurosciences that still lacks appropriate brain templates is the adult Yucatan micropig. In particular, T2-weighted templates are absent in this species as a whole. To address this need and provide a tool for neuroscientists wishing to pursue neuroimaging research in the adult micropig, we present the construction of population averaged (*n* = 16) T2-weighted MRI brain template for the adult Yucatan micropig. Additionally, we present initial analysis of T1-weighted (*n* = 3), and diffusion-weighted (*n* = 3) images through multimodal registration of these contrasts to our T2 template. The strategies used here may also be generalized to create similar templates for other study populations or species in need of template construction.

## Introduction

The Yucatan micropig (*Sus scrofa domesticus*) is an important and useful large animal model in translational neuroscience, given the similarities in size, anatomy, and physiology between the brains and spinal cords of pigs and humans (Lind et al., 2007; Bjarkam et al., 2008; Sauleau et al., 2009; Lee et al., 2013; Noga et al., 2020). Whereas the rodent model has scientific advantages based on the availability of transgenic and optogenetic toolkits, many neuroscientists recognize its limitations as a clinical model (STAIR, 1999; Kwon et al., 2010, 2013). Non-human primates (NHP) have long been considered the gold standard for clinical neuroscience research; however, the significant ethical and financial barriers associated with NHP research are prohibitive to many investigators (Garbarini, 2010; Prescott, 2010). Thus, the laboratory pig has emerged as a practical alternative model for many neuroscientists. While the domestic pig's fast growth rate and large overall size (>100 kg by 4 months age) can also present researchers with undue logistical and financial challenges related to animal handling and providing adequate pen-housing, animal feed, and weight-control, several miniature breeds have been developed to mitigate this issue (Swindle et al., 2012). The most common miniature breeds used in the US, in ascending order of size (at sexual maturity), are the Göttingen (10–14 kg), Yucatan micropig (14–20 kg), Sinclair (16–22 kg), Yucatan minipig (20–30 kg), and Hanford (25–40 kg) (Smith and Swindle, 2006). Although they continue to grow throughout adulthood, the relatively small size of the Yucatan micropig and its docile temperament make for generally lower maintenance costs and easy handling.

**Figure 1:**
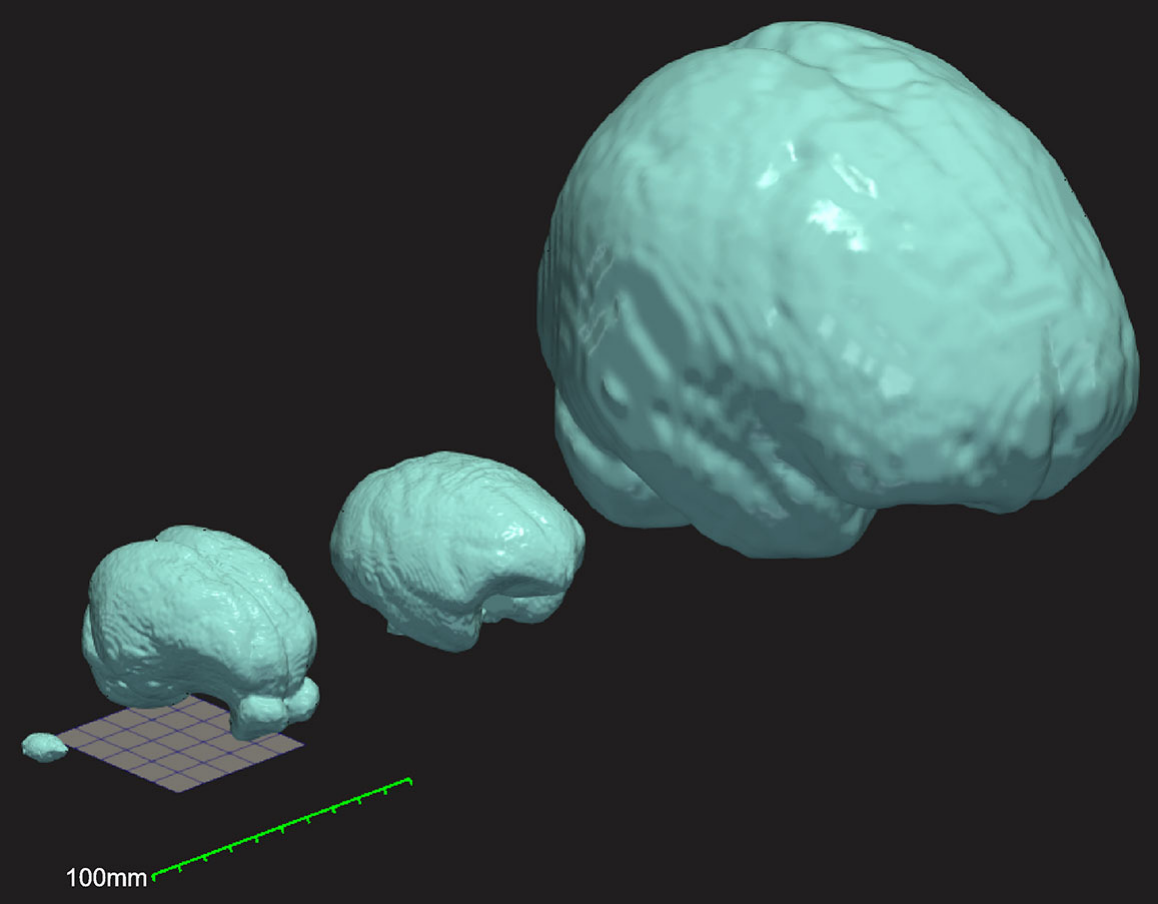
Three-dimensional comparison of brain size and morphology for mouse, Yucatan micropig, macaque, and humans (left to right). Surface mesh reconstructions of each brain were created from publicly available brain templates for the C57Bl6 mouse (Hikishima et al., 2017), the Yucatan micropig (this paper), the macaque (Rohlfing et al., 2012), and humans (Fonov et al., 2009), in ITK-Snap and visualized in ParaView. A 50 mm × 50 mm grid and a 100 mm ruler are displayed to show scale.

The large size of the micropig brain makes it possible to obtain *in vivo* neuroimaging in these animals with widely available conventional CT and MRI machines designed for humans (Sauleau et al., 2009) (Figure 1). While this feature greatly expands the potential experimental versatility and accessibility of this model, appropriate standardized reference templates and *a priori* tissue probability maps are required to fully realize this potential and facilitate the automated and unbiased processing of neuroimaging data in the Yucatan micropig (Ashburner and Friston, 2000, 2005). Numerous such templates have been developed over the years for this purpose in humans (Fonov et al., 2009), non-human primate species (Quallo et al., 2010; Frey et al., 2011), dogs (Nitzsche et al., 2019), cats (Stolzberg et al., 2017), sheep (Nitzsche et al., 2015), and rodents (Bai et al., 2012; Papp et al., 2014). Several MRI-based brain atlases have been created for the pig model (Watanabe et al., 2001; Saikali et al., 2010; Conrad et al., 2014; Gan et al., 2014; Zhong et al., 2016), although with important limitations for the abovementioned use case. Most previous templates have been created in neonatal piglets (3–6 weeks old) for use as a neurodevelopmental model (Conrad et al., 2014; Gan et al., 2014; Zhong et al., 2016), with significant morphological differences to the sexually-mature pig brain (Conrad et al., 2012; Zhong et al., 2016). Saikali et al. (2010) created a high-resolution three-dimensional brain atlas, albeit using a single 6-month-old pig and thus limiting its generalizability in analyzing a population of animals. Furthermore, this atlas is no longer freely available and requires software purchase to access. Finally, Watanabe et al. (2001) created a population averaged T1 template from 22 male adult Göttingen minipigs (9–11 months); however, this template was limited to linear registration methods available at the time, which produce blurry averages (Seidlitz et al., 2018). Currently available tools employ non-linear deformation fields for normalization of individual images as part of an unbiased iterative averaging process that produce improved detail and contrast (Avants et al., 2010). Furthermore, the Watanabe and Saikali brain templates are T1-weighted, whereas diffusion-weighted imaging (DWI) provides important additional information about white matter organization, and T2-weighted MRI can provide enhanced visualization of several important deep brain structures owing to their iron content (red nucleus, substantia nigra pars reticulata, globus pallidus) (Lalys et al., 2010; Telford and Vattoth, 2014; Pai et al., 2020).

In this manuscript, we describe the generation of an *in vivo*, population averaged, T2-weighted MRI-based stereotaxic brain template for the adult Yucatan micropig. This data is presented in the Neuroimaging Informatics Technology Initiative (NifTI) format, for convenient use and incorporation into popular neuroimaging analysis toolkits (FSL, SPM, Slicer, ANTs). These datasets will thus contribute to the processing and analysis of brain imaging data in adult micropig research.

## Materials and Methods

### Study Population

Cranial imaging data was acquired from 16 healthy and neurologically normal female adult Yucatan micropigs (age 6–25 months; Sinclair BioResources, LLC), as baseline imaging for research purposes prior to any experimentation (Table 1). Animals were housed either individually or in pairs in temperature-controlled pens and fed twice a day, with access to water ad libitum. All animal research activities were approved by our local Institutional Animal Care and Use Committee (IACUC 18-033).

**Table 1:** Age and body weight data of the pigs used for template construction.

**Animal**	**Age (weeks)**	**Body weight (kg)**	**Imaging modalities**
1	33	26	T2
2	25	24	T2
3	27	24	T2
4	28	22	T2
5	36	23	T2
6	103	58	T2
7	57	51	T2
8	68	58	T2
9	28	23	T2
10	30	26	T2
11	27	25	T2
12	27	24	T2
13	26	22	T2
14	27	24	T1, T2, DWI
15	31	26	T1, T2, DWI
16	32	27	T1, T2, DWI
Mean	39.6	31.2	–

### Image Acquisition

All subjects were imaged under general anesthesia, with intubation and mechanical ventilation. Anesthesia was induced with an intramuscular mixture of telazole (8 mg/kg) and xylazine (1.5 mg/kg) and maintained with isofluorane (2%) through the endotracheal tube once intubated. Subjects' vitals were monitored using an MRI compatible pulse oximeter and ECG and temperature probes (Tesla 3M monitor, Germany), and subjects were positioned prone on the table with a Bair Hugger™ (3M™) warming pad over the body to help maintain body temperature. A high-resolution, single-slab, three-dimensional (3D) isotropic, T2-weighted turbo-spin-echo (TSE) Sampling Perfection with Application optimized Contrasts using different flip angle Evolution (SPACE) sequence was performed in the coronal plane. This was accomplished using a Siemens 3T Trio MRI scanner (Siemens, Erlangen, Germany) with Syngo MR Software (Numarus 4, VB-19), a 16-channel body surface coil (Invivo, Gainsville, Florida, USA) and the table spine coil elements to obtain optimal signal uniformity. Sequence parameters were as follows: repetition time (TR) = 2,050 ms; echo time (TE) = 128 ms; flip angle = 120°; matrix = 320 × 320; field of view (FoV) = 160 mm × 160 mm; slice thickness = 0.5 mm, imaging time = 19 min.

In addition to the T2-weighted imaging performed in all subjects, T1-weighted and diffusion-weighted imaging were performed in 3 subjects (Table 1). For T1-weighted imaging, a 3D magnetization prepared gradient echo (MPRAGE) sequence was performed in the axial plane, with the following sequence parameters: TR = 1,900 ms, TE = 3.39 ms, flip angle = 9°, matrix = 224 × 256, FoV = 175 mm × 200 mm, slice thickness = 0.7 mm, imaging time = 7 min. Diffusion-weighted imaging was acquired using an echo-planar spin-echo (EPSE) sequence, with TR = 3,300 ms; TE = 93 ms; flip angle = 90°; slice thickness/spacing = 3.00/3.00 mm; matrix = 128 × 128; FoV = 200 mm × 200 mm; with b-value (1) baseline image of 0 s/mm^2^ and b-value (2) of 1,000 s/mm^2^ along 30 directions; EPI factor 128, imaging time = 9 min.

### Data Preprocessing

**Figure 2:**
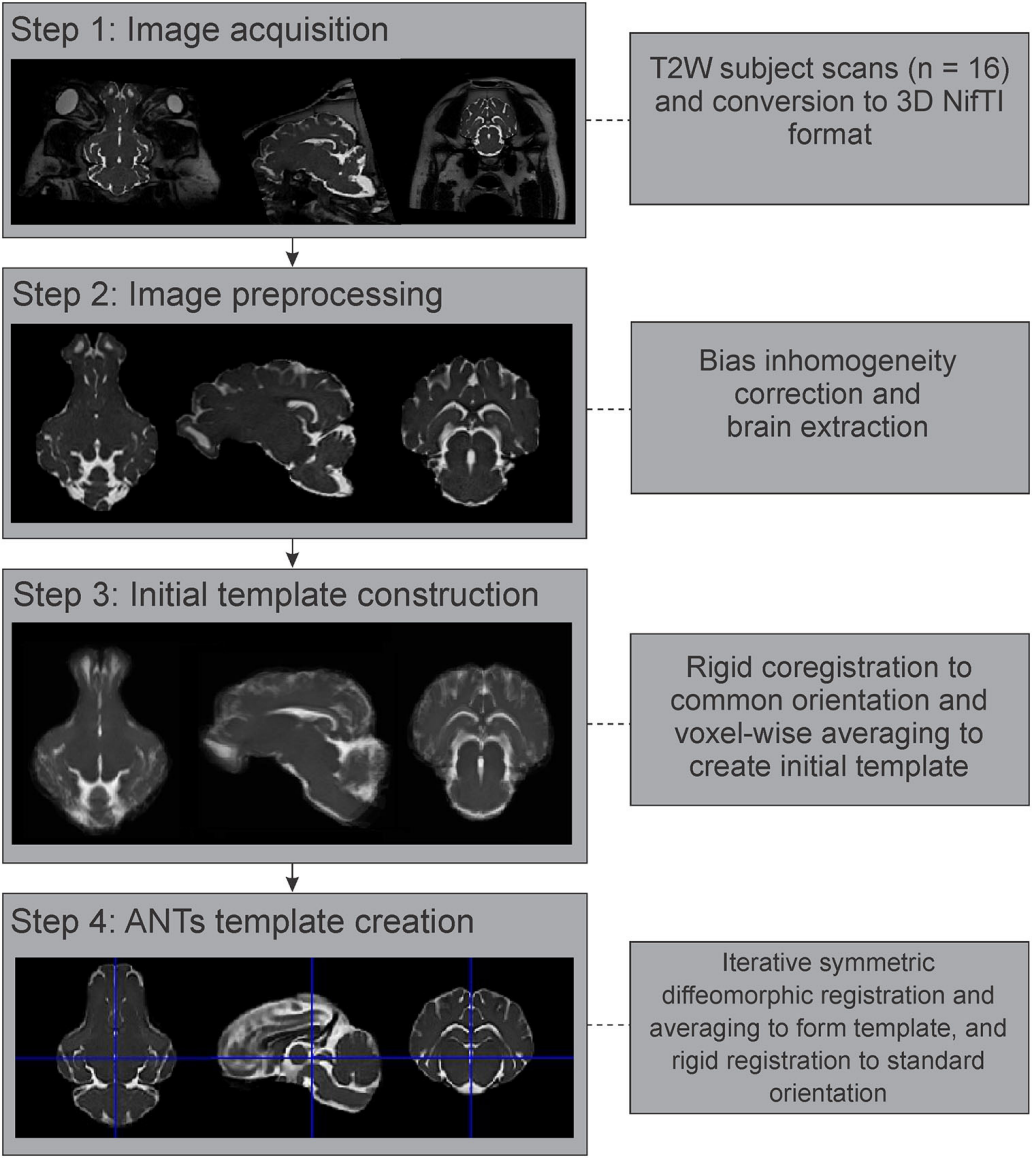
Image processing and template generation pipeline. The major steps and processes that were involved in generating the population averaged T2 brain template are outlined. NifTI, Neuroimaging Informatics Technology Initiative; ANTs, advanced normalization tools.

All individual images were converted from Digital Imaging and Communications in Medicine (DICOM) format to the NifTI format using dcm2niix (Li et al., 2016), prior to further processing (Figure 2). These images were then corrected for intensity inhomogeneity using the N4BiasFieldCorrection tool from the Advanced Normalization Tools (ANTs) suite (Tustison et al., 2010; Avants et al., 2011). As pigs have significant bone and extra-cranial tissues that could affect image processing steps, brain masks were generated for each individual image using the Brain Extraction Tool (BET2) from the FMRIB Software Library v.6.0 (FSL) (Analysis Group, Oxford, UK; https://fsl.fmrib.ox.ac.uk/fsl/fslwiki/FSL) (Jenkinson et al., 2012). These automatically generated brain masks were manually corrected and optimized using 3D Slicer v.4.10.2 (https://www.slicer.org/) (Fedorov et al., 2012), prior to brain extraction. Diffusion-weighted images were further preprocessed using the *denoise* and *dwifslpreproc* functions in MRtrix3 (https://www.mrtrix.org/) (Smith et al., 2004; Veraart et al., 2016a,b; Cordero-Grande et al., 2019; Tournier et al., 2019).

### T2 Template Construction

The preprocessed and brain extracted T2 images from 16 adult Yucatan micropigs were used to generate a population averaged template, with the major steps outlined in Figure 2. As previous atlas literature suggests that non-linear methods of template construction result in better signal- and contrast-to-noise ratios than linear methods (Klein et al., 2009), we decided to use the highly ranked Advanced Normalization Tools (ANTs) for our template generation (Avants et al., 2011). All 16 images were linearly registered to a randomly selected individual image in SPM12, prior to calculating the population mean (Rigid template). This was used as the initial template for the ANTs symmetric normalization (SyN) algorithm, through the ANTs multivariate template construction script (*antsMultivariateTemplateConstruction2.sh*), which iteratively transformed the 16 images into a common space using rigid, affine, and symmetric diffeomorphic registration (Avants et al., 2011). This template was reoriented in SPM12 to match the stereotaxic coordinate system used by precedent pig atlases, with the origin set to the anterior edge of the posterior commissure at the midline, with the plane of the origin extending through the centers of both the anterior and posterior commissures in the midline, and its orthogonal vector centered within the midsagittal plane.

### Example T1 Template Construction

**Figure 3:**
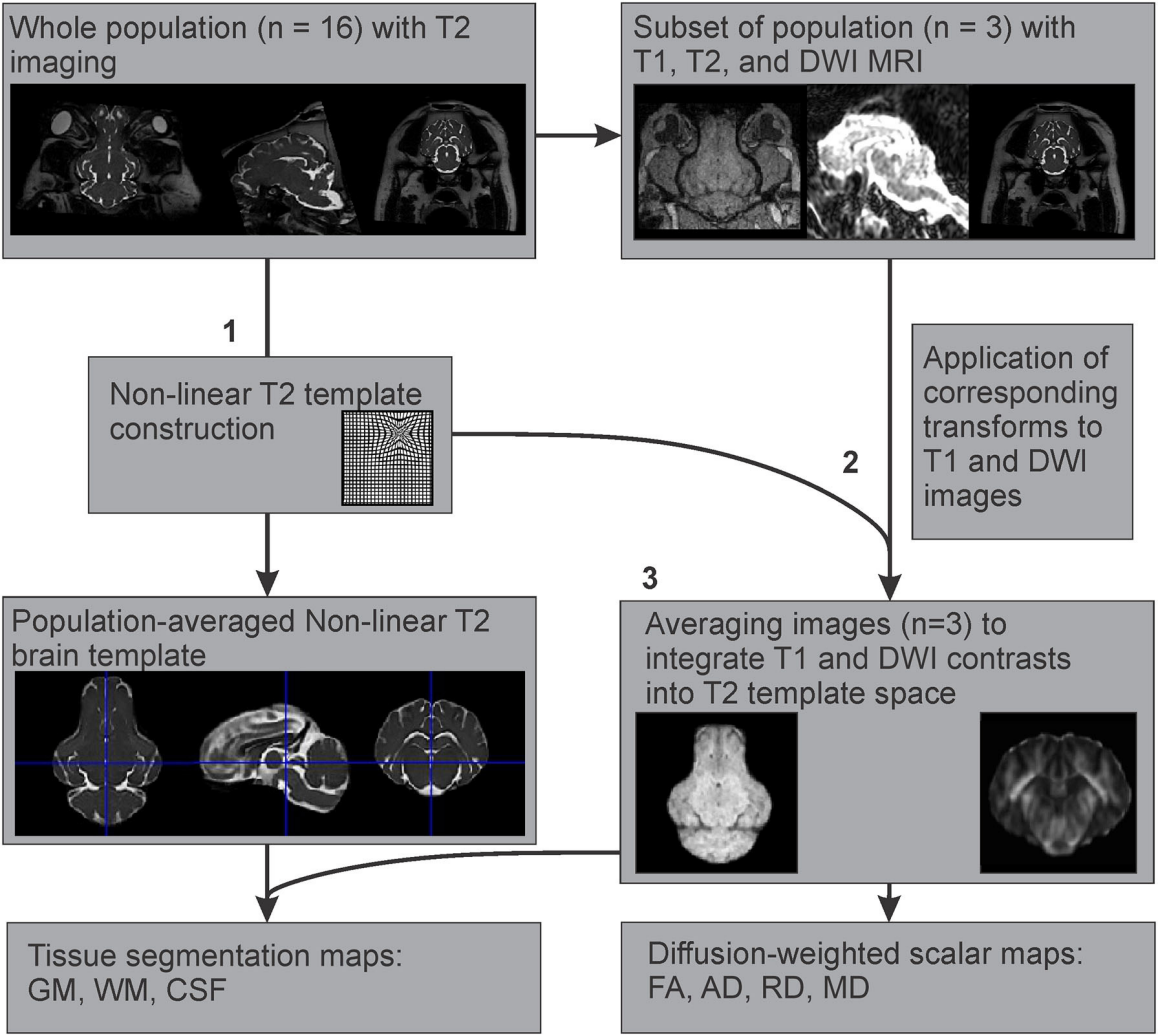
Multimodal registration of T1 and diffusion-weighted templates to the T2-weighted template space. After **(1)** creation of the population averaged T2 template, **(2)** the generated non-linear transformations were applied to the corresponding T1 and DWI images from a subset of the population where all three imaging modalities were acquired, to map their features onto the T2 template space. **(3)** These images (*n* = 3) were averaged to create T1 and DWI templates based on the T2 population template.

Since T1 images were only available for three individuals, an example T1 template was created using multimodal registration of these images to our population averaged T2 template. The preprocessed and brain extracted T1 images available from three adult Yucatan micropigs were warped into the T2 template space, by applying the transformations generated during the construction of our T2 non-linear template from the corresponding subjects. These warped T1 images were then averaged using the ANTs multivariate template construction script to create an example T1 template, based on our population averaged T2 template (Figure 3).

### Structural Template Quality Assessment

The quality of the generated T2 template was gauged by calculating gray matter and white matter signal-to-noise ratios (SNR) and contrast-to-noise ratios (CNR) using Equations (1, 2),

(1) $$\begin{array}{r} SNR_{ROI}=\frac{{\bar{G}}_{ROI}}{\sigma_{ROI}} \end{array}$$

(2) $$\begin{array}{r} CNR_{GM/WM}=\;\frac{\left|SNR_{GM}\;-\;SNR_{WM}\right|}{\sqrt{\sigma_{GM}^{2}+\sigma_{WM}^{2}}} \end{array}$$

where ${\bar{G}}_{ROI}$ is the mean gray value for the voxels in a region of interest (ROI), and σ is the standard deviation for that set of gray values. SNR for gray matter was calculated over spherical ROIs (radius = 1 mm, 31 voxels) in the caudate nucleus (*n* = 8), while SNR for white matter was calculated over ROIs in the corpus callosum in the midline (*n* = 8). These values were compared to identical calculations performed for a sample T2 individual image, as well as the initial T2 template generated using linear registration methods. The data were tested for normality using Shapiro-Wilk test, with no relevant deviations from normality found. A one-way analysis of variance (ANOVA) test was used to analyze differences in GM SNR, WM SNR, and CNR between images (individual sample, rigid template, non-linear template).

Landmark validation was performed using the anterior commissure (AC) and posterior commissure (PC). The centers of these landmarks were selected manually for each subject co-registered to the T2 template space, before comparing their spatial locations with the template to calculate the landmark variation. Mean (±SD), maximum, and minimum distances were calculated for both landmarks.

### Tissue Probability Map Construction

The T2 and T1 templates were used to generate a tissue probability map (TPM) using the FMRIB Automated Segmentation Tool (FAST) from the FSL suite (Analysis Group, FMRIB, Oxford, UK) (Zhang et al., 2001). Parameters were set to segment the template into four different binary tissue class masks: gray matter (GM), white matter (WM), cerebrospinal fluid (CSF), and other (extracranial) remnants. These tissue masks were then manually corrected to better fit the anatomy. FAST was also used to generate partial volume tissue maps, which were used to calculate mean tissue volumes and ratios (Table 2).

**Table 2:** Brain template tissue segmentation volumes and landmark variation.

**Tissue volumes (mL)**
Gray matter			56.9
White matter			46.0
Cerebrospinal fluid			31.1
Total brain			102.9
**Volume ratios**
GM:WM ratio			1.2
GM:total brain ratio			0.6
CSF:total brain ratio			0.3
**Landmark variation**	**Mean** **±** **SD (mm)**	**Max (mm)**	**Min (mm)**
Anterior Commissure	0.58 ± 0.27	1.00	0
Posterior Commissure	0.58 ± 0.42	1.12	0

### Example DWI Template Construction and Tractography

The preprocessed diffusion-weighted images from the same three individuals as in the T1 template generation were used to estimate diffusion tensors using the *dwi2tensor* function in MRtrix3 (Basser et al., 1994; Veraart et al., 2013). The diffusion tensors were then used to calculate the following scalar maps: fractional anisotropy (FA), axial diffusivity (AD), mean diffusivity (MD), and radial diffusivity (RD). As the B0 images are less susceptible to eddy-current and motion distortions, they were extracted from each DWI dataset to use for intra-subject ANTs SyN registration to the T2 structural image. These transformations were then applied to each scalar map to correct for susceptibility-induced distortions. Then the corresponding transformations generated during the T2 non-linear template construction were applied to the appropriate scalar maps, before averaging these using the ANTs multivariate template construction script, to emulate DWI templates based on our population averaged T2 template (Figure 3).

Whole brain tractography was performed using MRtrix3 in a single individual with the highest quality DWI scan, which was warped into the T2 template space. The brain mask used for the structural MRIs was manually adapted for the DWI. The response function for fiber tracts was estimated in voxels with a FA value > 0.3 using a method that better resolves crossing fibers (Tournier et al., 2007; Tax et al., 2014). Fibers were generated using the SD-Stream algorithm, using parameters described previously (10,000 fibers, 0.1 mm step size, cutoff length of 0.2 mm, minimum length 5 mm) (Zhong et al., 2016). The tractography data was converted to a .vtk file to allow for 3D reconstruction and was overlaid in a 3D reconstruction of the T2 brain template for visualization.

## Results

### Study Population Characteristics

All 16 animals were healthy and neurologically normal at the time of imaging, and no animal had to be excluded from the study. Subject ages and weights at the time of imaging, and the imaging modality acquired are shown in Table 1.

### Structural Template Quality Assessment

**Figure 4:**
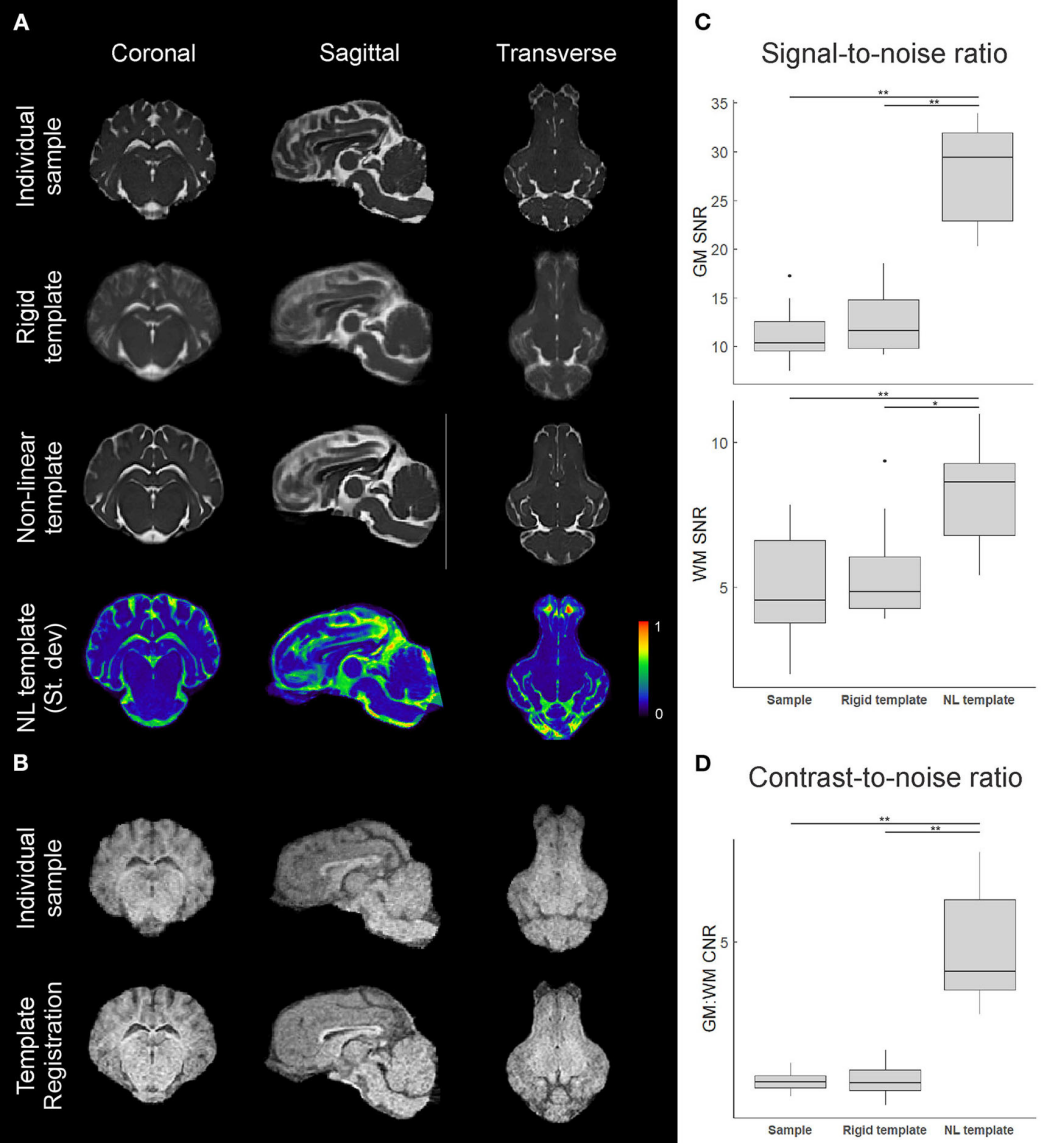
Comparison of individual and template datasets. **(A)** T2-weighted datasets registered to the common stereotactic space for visual comparison between an individual sample, the rigid-body co-registration population average (rigid template), and the non-linearly transformed brain template. Mid-coronal, mid-sagittal, and mid-transverse slices are shown. A 3D plot of the normalized voxel-wise standard deviation for the T2 non-linear template is also shown, with a slightly lower transverse slice shown to visualize the olfactory bulb. Areas in red indicate relatively higher inter-subject structural variability. **(B)** Similar visualizations comparing an individual sample T1 image and the T1 template (*n* = 3) formed through multimodal registration to the T2 template space. **(C)** Calculated gray matter (GM) and white matter (WM) signal-to-noise ratios (SNR) for an individual image, the rigid template, and the non-linear template, based on caudate nucleus and corpus callosum sampling. **(D)** Calculated gray matter to white matter contrast-to-noise ratios (CNR) for each image based on the SNRs from **(C)**. Boxplots show median, 25/75% quartiles, and whiskers extending to the smallest or largest value no further than 1.5 times the interquartile range from the hinge. Points beyond this range are shown as outlier dots. *p < 0.05, **p < 0.01.

In addition to revealing morphological characteristics of the brain at a population level, averaging images generally reduced the standard deviation of voxel gray values within structures, which improves SNR and CNR; however, with the rigidly co-registered images, the average template had blurred edges and borders which canceled much of these benefits (Figures 4A,B). The non-linearly co-registered and averaged template had sharper edges and increased resolution when visually compared to the individual and rigid template images. The GM and WM SNR in the T2 non-linear template were significantly improved compared to the individual and rigid template images (*p* < 0.05; Figure 4C). Finally, the GM:WM CNR was significantly improved in the T2 non-linear template compared to the individual and rigid templates (*p* < 0.01; Figure 4D). Areas of structural variability between subjects in the population were revealed by calculating the voxel-wise standard deviation of gray values for the T2 non-linear template (Figure 4A). Most gray and white matter structures had low variability in the non-linear template, with most of the inter-subject variability being in CSF structures, particularly within the olfactory bulb. Landmark distance variations between the individual images and the template were within 1 mm for the anterior commissure, and 1.12 mm for the posterior commissure, and averaged 0.58 mm (~1 voxel) for both of these landmarks in the population (Table 2).

### Tissue Segmentation Maps, Brain Volumes, and Cortical Surface Architecture

**Figure 5:**
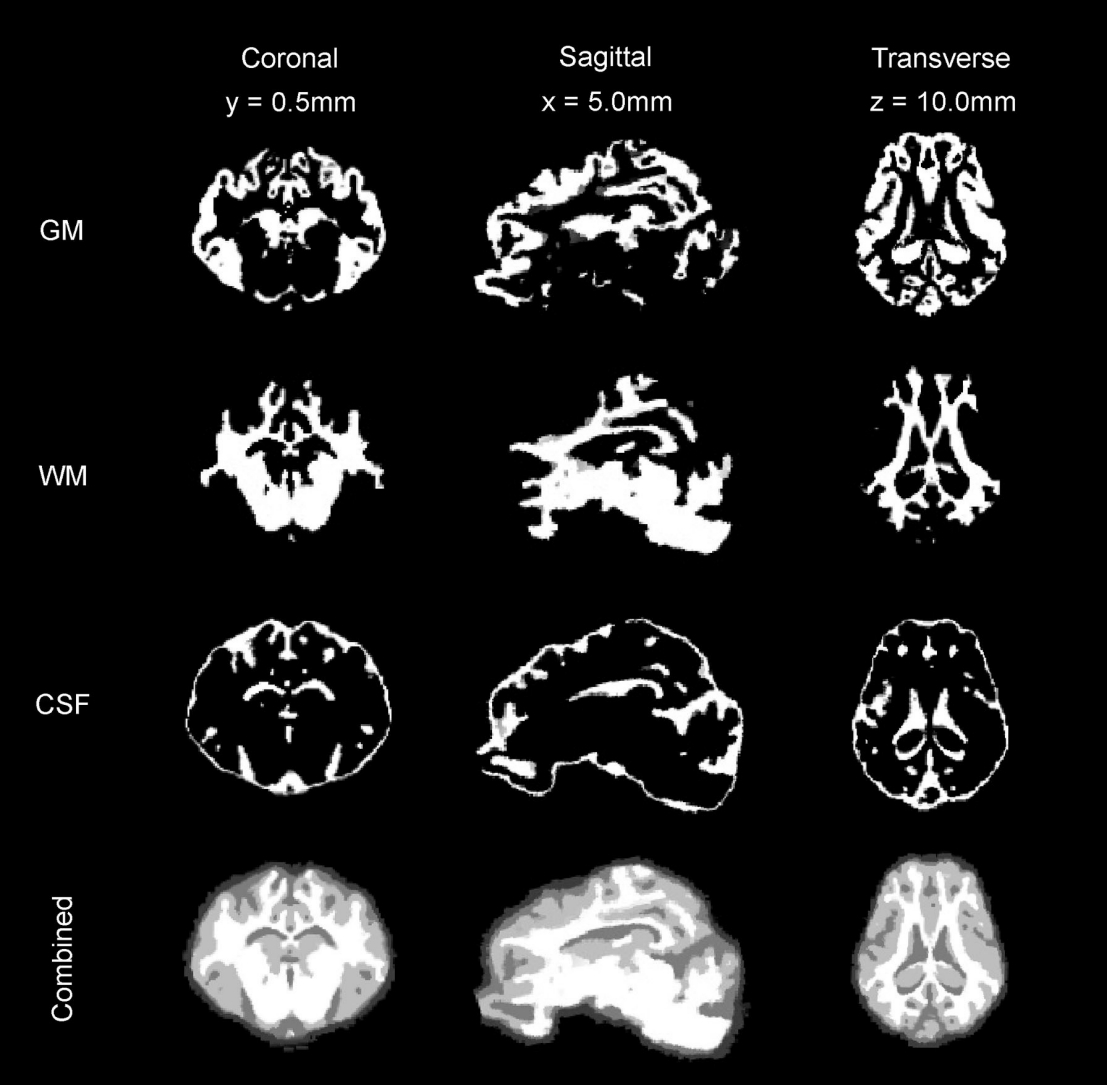
Tissue segmentation maps. The gray matter (GM) map, white matter (WM) map, and cerebrospinal fluid (CSF) maps generated from the T2 and T1 templates are presented in coronal, sagittal, and transverse planes.

**Figure 6:**
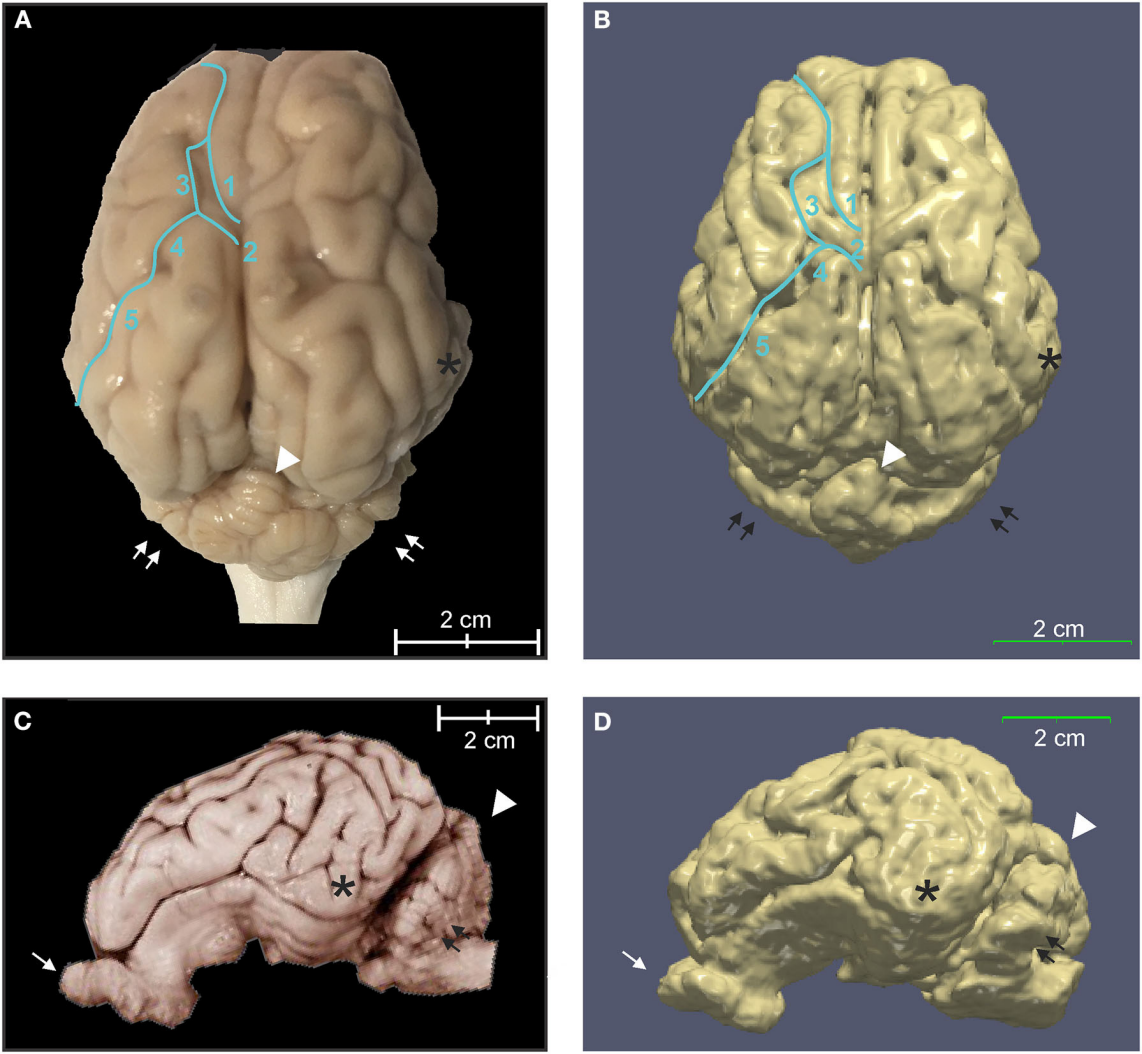
Three-dimensional rendering of cortical surface architecture compared to fixed specimen. **(A)** Dorsal and **(C)** side views of fixed specimens are compared with corresponding 3D renderings **(B,D)**. 1: cruciate sulcus, 2: ansate sulcus; 3: coronal sulcus; 4: connection sulcus; 5: median suprasylvian sulcus. Asterisk (*), temporal lobe; arrow, olfactory bulb; double arrows, cerebellar hemispheres; arrowhead, cerebellar vermis.

The binary tissue probability maps for GM, WM, and CSF were calculated using both the T2 and T1 templates (Figure 5), and can be used as a mask for brain extraction, or as an *a priori* input for tissue segmentation of adult micropig brains in programs such as SPM12 or FSL. The partial volume maps for GM and WM were used to compute CSF, as well as gray matter and white matter tissue volumes in the T1 template, to increase accuracy (Table 2) (Johnson et al., 2019). Cortical surface architecture was estimated by concatenating GM and WM masks, and then creating a surface mesh 3D object from the volume. The surface architecture of the 3D reconstruction showed similar features when compared to a fixed gross brain specimen (Figure 6).

### Example DWI Template and Whole Brain Tractography

**Figure 7:**
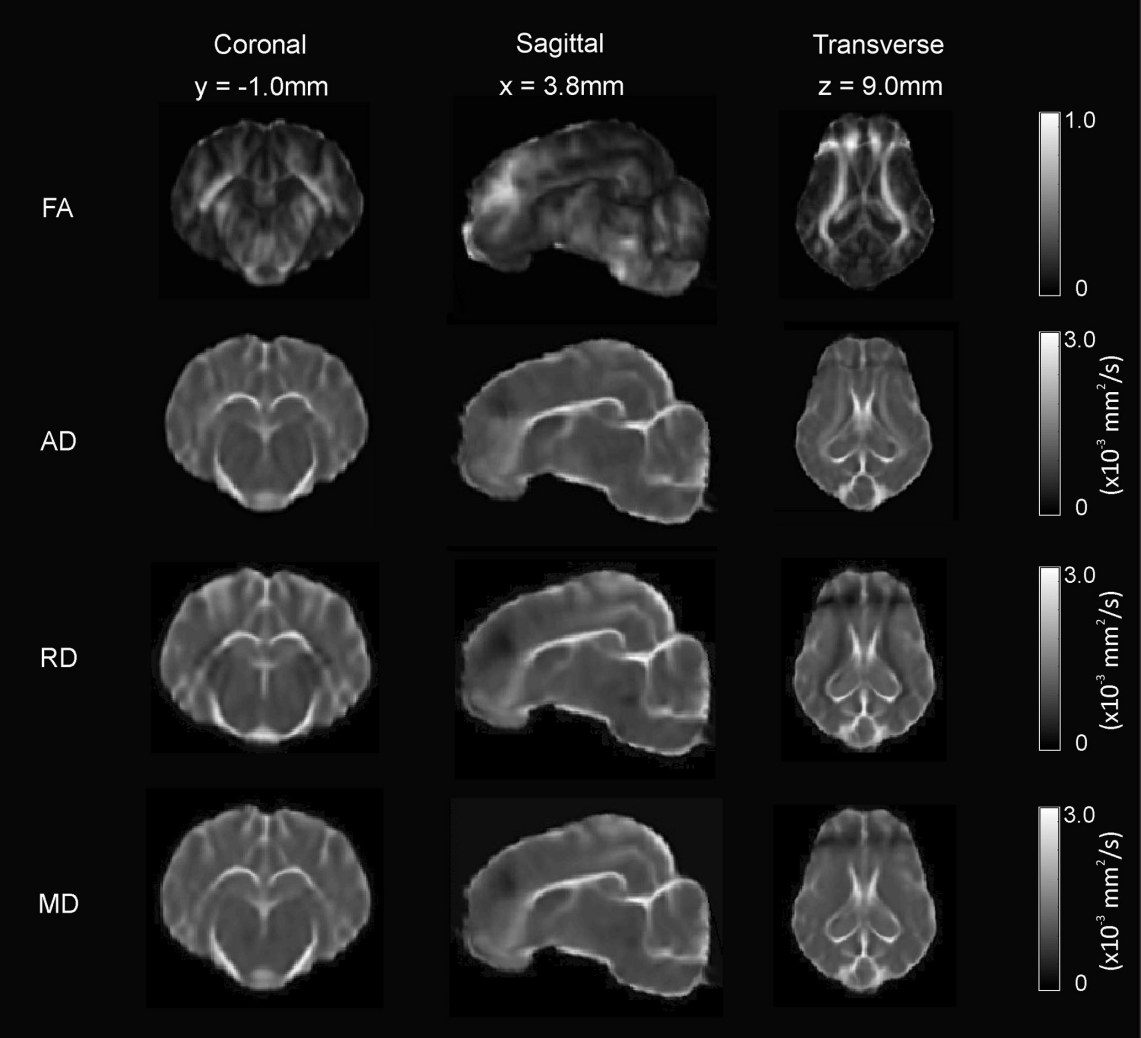
Diffusion-weighted scalar template maps. The fractional anisotropy (FA), anisotropic diffusion (AD), mean diffusivity (MD), and radial diffusivity (RD) template maps are presented in coronal, sagittal, and transverse planes.

**Figure 8:**
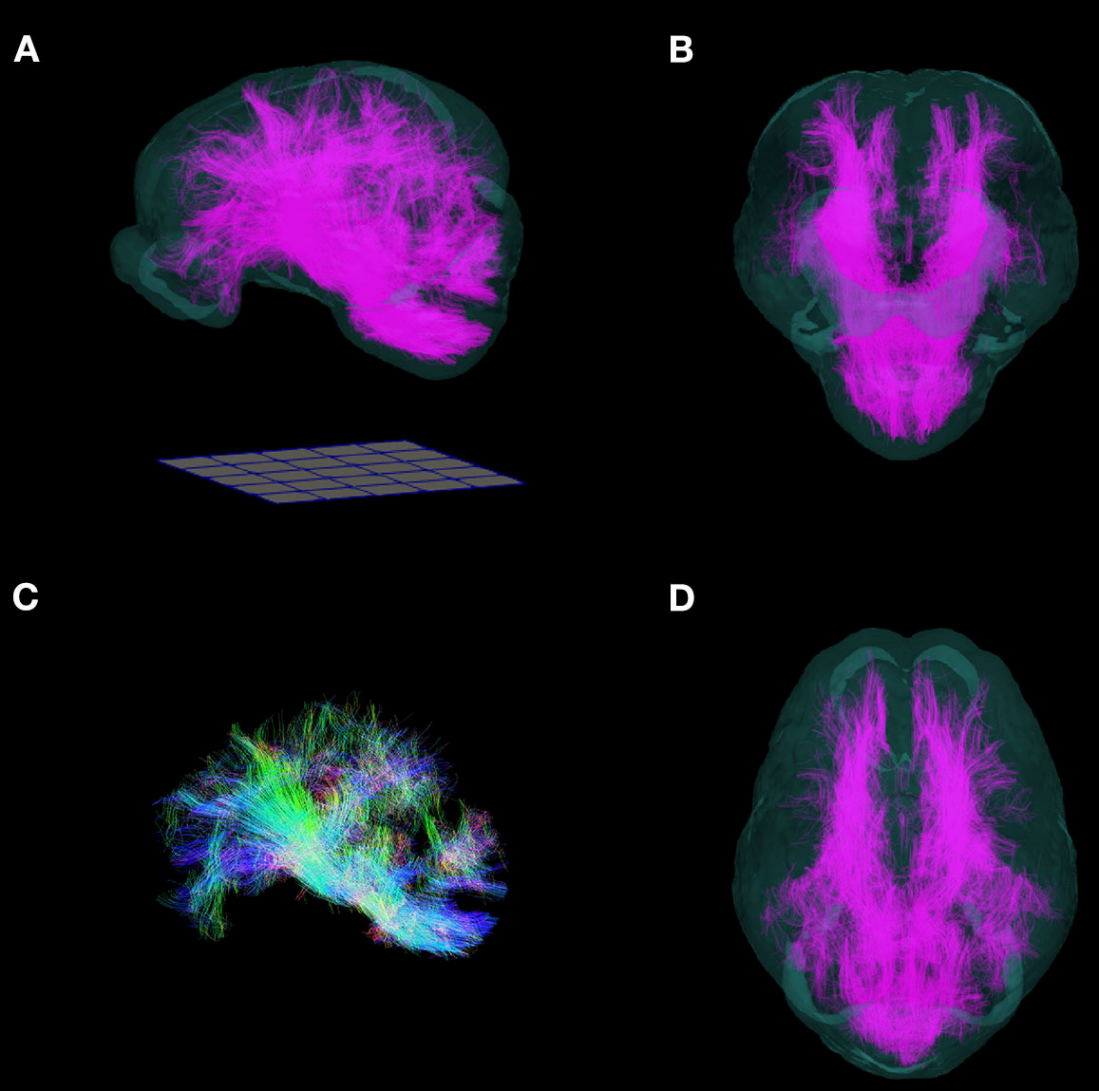
Three-dimensional reconstruction of whole brain tractography in the pig. 3D reconstruction of white matter fiber tracts warped into the T2 template space are superimposed into a translucent rendering of the brain template, and shown in **(A)** side, **(B)** frontal, **(D)** and top views. **(C)** Whole brain tractography without the brain template. (Fibers directed along the antero-posterior axis are blue, supero-inferior axis: green; right-left axis: red). Renderings were implemented in ParaView. A 50 mm × 50 mm grid is shown for scale.

The FA, AD, RD, and MD scalar map example templates produced for the population are presented in Figure 7. High FA values were present in large, well-defined white matter tracts, and this was reflected to a lesser degree in the AD map as well. The RD map showed low signal in these same white matter tracts. Overall, the AD, RD, and MD maps demonstrated highest signal in areas of CSF. Whole brain tractography from the individual with the highest quality scan is presented in Figure 8 as a 3D reconstruction superimposed on to the brain template.

## Discussion

### The Pig Model in the Neurosciences

The relatively large and convoluted pig brain makes it an excellent model for study using neuroimaging techniques, including with commonly available medical-grade scanners. This study presents the first T2-weighted population averaged MRI brain templates for the adult Yucatan micropig, using non-linear registration methods. It is oriented in the standard stereotaxic space, which will allow simple compatibility with other pig templates and atlases, including prior parcellation maps. These templates, along with the tissue probability maps are freely available online (www.nitrc.org/projects/micropig_brain) and provide researchers with population-level information on the morphological and structural characteristics of the adult micropig brain, and serve as useful and standardized tools to process and analyze pig brain imaging datasets. Normalizing individual datasets to this population averaged template increases the generalizability of those datasets and allows for comparisons to be made between individuals at the group level. This is useful for many fields of study in neuroscience, such as in fMRI studies, where regions of activation need to be correlated to anatomic structures, and stereotaxic electrode studies, where the locations of electrodes need to be extrapolated to a standardized space. The pig model is well suited to both examples, with a few important considerations discussed below, and our templates and tools would be of benefit in these circumstances.

While the size of the pig brain stabilizes after sexual maturity, making brain masking relatively straightforward with the use of brain templates such as ours, the skull undergoes significant changes throughout adulthood that can complicate experiments and surgical procedures in the pig. In the Yucatan micropig, the skull both thickens and pneumatizes due to the frontal sinus with age, such that the calvarial thickness at the level of the bregma can vary from 5 mm in the 25 week old pig, to over 20 mm in the 100 week old pig. This can make surgical access and post-mortem extraction of the brain quite challenging in this animal, as has been noted for the Göttingen minipig (Sauleau et al., 2009). Furthermore, in contrast to the rodent model, where stereotaxic apparatus often uses the auditory canals, along with the hard palate as fixation points for the skull, the oblique angle of the auditory canal in the pig necessitates the use of other points of skull fixation for stereotaxic equipment, such as the zygoma (Sauleau et al., 2009).

### The T2-weighted Yucatan Micropig Brain Template

Our use of non-linear template construction methods resulted in a sharp image with high SNR and CNR, compared to individual images or brain templates created using only linear methods, as was used for the Göttingen minipig template (Watanabe et al., 2001; Andersen et al., 2005). Furthermore, the inclusion of TPMs further extends the utility of our toolkit, providing researchers with an easy way to automatically segment or extract adult micropig brains from raw neuroimaging data of different modalities. T1 and DWI templates are provided from images of three subjects, applying multimodal registration to our T2 template; however, these cannot be considered population averaged templates themselves, as they are based on images from only three individuals. Rather, they are better considered an integration of T1 and DWI contrast features onto our T2-weighted population averaged template.

Recently, a preprint for a paper detailing the construction of a T1-weighted population averaged template in adult male Yucatan minipigs has been made available online through the bioRxiv server by an independent group (Norris et al., 2020). This timely contribution speaks to the increasing interest in miniature pigs as a neuroscience model, and complements our contribution nicely, since our template is T2-weighted and constructed from female adult micropigs. Although Yucatan minipigs tend to be larger than the micropig variety, the pigs in Norris et al. were imaged earlier (mean 5.5 vs. 10 months in our study) and were thus smaller (mean weight 23 vs. 31 kg in our study). The template in Norris et al. uses 70 subjects compared to 16 in our study; however, the image acquisition in that study was noted to be resolution limited at 1 × 1 × 1 mm^3^, compared to 0.5 × 0.5 × 0.5 mm^3^ in our study. Both studies used non-linear template generation methods and compared them to linear registration methods, although Norris et al. used AFNI (Cox, 1996), while our study used the Advanced Normalization Tools (Avants et al., 2011). Co-registration and visual comparison of these two templates show significant similarity in size and structure, with our template having increased resolution. A comparison of landmark variation measurements for the AC and PC show that our template had smaller errors (0.58 mm vs. ~1 mm), and this is likely due to the increased resolution of our template. Similarly ranged values for landmark variation have been produced in other pig brain templates, including two neonatal piglet templates (0.41 and 0.65 mm for AC and PC, respectively) (Conrad et al., 2014), (0.85 and 0.72 mm) (Zhong et al., 2016).

The tissue volumes calculated from our brain template in Table 2 indicate the adult brains used in our study were at least 50% larger than those of piglets (Zhong et al., 2016). The GM:WM ratio was lower in the micropig (1.2) compared to those published for the ovine brain template (1.45) (Nitzsche et al., 2015), and the canine brain template (1.85) (Nitzsche et al., 2019), while the GM:total brain volume was similar among these three species (~0.60).

### Limitations

The main limitations of our population averaged brain template are the low subject number and the inclusion of only female subjects. We included 16 animals in generating the T2 brain template based on the observation that templates stabilize at around 10 subjects (Avants et al., 2014) and the numbers used in other similar studies (Zhong et al., 2016; Villadsen et al., 2018; Johnson et al., 2019); however, we recognize that many human brain templates include one hundred or more subjects, and larger numbers would increase our confidence that our template is representative of the population. All the animals used in our study were female as we work exclusively with female micropigs. While GM/WM composition differences between male and female brains are described for humans (Allen et al., 2003), no significant difference was reported in sheep (Nitzsche et al., 2015). Thus the exact magnitude of this limitation is uncertain in our study; rather, other factors such as age appear to play a bigger role in determining important morphological differences (Nitzsche et al., 2015; Johnson et al., 2019; Liu et al., 2020). Finally, although our template is representative of the inter-individual variation found amongst the adult Yucatan micropig population, this variation is likely different for other pig breeds, including other miniature breeds. Thus, our template is most appropriately applied within adult Yucatan micropig, and potentially minipig populations, and would likely have diminishing accuracy in application to other pig populations, especially those differing significantly in size from the Yucatan micropig.

## Data Availability Statement

The datasets presented in this study can be found in online repositories. The names of the repository/repositories and accession number(s) can be found in the article/Supplementary Material.

## Ethics Statement

The animal study was reviewed and approved by University of Miami Institutional Animal Care and Use Committee (IACUC 18-033).

## Author Contributions

SC: conceptualization, methodology, formal analysis, visualization, and writing – original draft. BN and SC: writing – review and editing. BN and JG: funding acquisition. All authors: investigation and data collection.

## Conflict of Interest

The authors declare that the research was conducted in the absence of any commercial or financial relationships that could be construed as a potential conflict of interest.
